# Differential expression of IL-6/IL-6R and MAO-A regulates invasion/angiogenesis in breast cancer

**DOI:** 10.1038/s41416-018-0078-x

**Published:** 2018-04-26

**Authors:** Rashmi Bharti, Goutam Dey, Anjan Kumar Das, Mahitosh Mandal

**Affiliations:** 1 School of Medical Science and Technology, Indian Institute of Technology Kharagpur, Kharagpur, 721302 India; 2 Department of Pathology, Calcutta National Medical Collage, Kolkata, West Bengal 70014 India

**Keywords:** Tumour angiogenesis, Breast cancer

## Abstract

**Background:**

Monoamine oxidases (MAO) are mitochondrial enzymes functioning in oxidative metabolism of monoamines. The action of MAO-A has been typically described in neuro-pharmacological domains. Here, we have established a co-relation between IL-6/IL-6R and MAO-A and their regulation in hypoxia induced invasion/angiogenesis.

**Methods:**

We employed various in-vitro and in-vivo techniques and clinical samples.

**Results:**

We studied a co-relation among MAO-A and IL-6/IL-6R and tumour angiogenesis/invasion in hypoxic environment in breast cancer model. Activation of IL-6/IL-6R and its downstream was found in hypoxic cancer cells. This elevation of IL-6/IL-6R caused sustained inhibition of MAO-A in hypoxic environment. Inhibition of IL-6R signalling or IL-6R siRNA increased MAO-A activity and inhibited tumour angiogenesis and invasion significantly in different models. Further, elevation of MAO-A with 5-azacytidine (5-Aza) modulated IL-6 mediated angiogenesis and invasive signatures including VEGF, MMPs and EMT in hypoxic breast cancer. High grade invasive ductal carcinoma (IDC) clinical specimen displayed elevated level of IL-6R and depleted MAO-A expression. Expression of VEGF and HIF-1α was unregulated and loss of E-Cadherin was observed in high grade IDC tissue specimen.

**Conclusions:**

Suppression of MAO-A by IL-6/IL-6R activation promotes tumour angiogenesis and invasion in hypoxic breast cancer environment.

## Introduction

Breast cancer is a deadly disease affecting the women health worldwide. Despite the advanced research since previous decades, it is further required to explore the molecular landscape of cancer progression, angiogenesis and metastasis. A number of functional activities of regulatory proteins have been discovered in cancer associated patho-biological states including tumour angiogenesis/invasion. Monoamine oxidase A (MAO-A) is an enzyme encoded by MAO-A gene. MAO-A is responsible for degradation and catalyzation of endogenous neurotransmitters amines including phenylethylamine, tyramine, serotonin, norepinephrine and dopamine in central, as well as peripheral region of the body.^1^ Critical roles of MAO-A have been discovered in context to a number of neurological disorders. MAO-A and another form MAO-B are generally present in outer mitochondrial membrane.^2^ However, there is a lack of knowledge in the domain of MAO-A involvement in cancer progression, metastasis and angiogenesis. Previous report showed that, MAO-A level was significantly decreased in multiple cancer types including breast cancer^3,4^ compared to adjacent normal tissues. In another investigation, serum starved apoptosis was co-related with increased MAO-A, caspase and MAPK expression in human neuroblastoma cells.^5^ MAO-A plays a critical role in staurosporine induced apoptotic cell death via altering caspase, Bcl-2 and p38/MAPK in SH-SY5Y human neuroblastoma cells.^6^ Clorgyline which is a MAO-A inhibitor can suppress the apoptotic effect of serum starvation in melanoma cells.^7^ Overall, MAO-A expression is reduced in breast cancer.

Interleukin-6 (IL-6) is a cytokine mainly involved in a number of inflammatory conditions.^8^ Growing evidences suggested that this cytokine was also involved in cancer progression, metastasis, chemo-resistance, angiogenesis and epithelial to mesenchymal transition.^9^ IL-6 is associated with more aggressive and invasive phenotype of cancer. IL-6 acts as a growth factor and fuels cancer progression through activating a series of downstream signalling cascade including gp130, JAK/STAT, MAPK, and Akt.^10^

In this study, we found that decreased level of MAO-A promotes tumour angiogenesis and invasion in breast cancer model in hypoxic environment. The activity of MAO-A is found to be negatively regulated by IL-6/IL-6R. Increased MAO-A was found to cause inhibition of angiogenic and invasive features of breast cancer cells. Inhibition of IL-6/IL-6R signalling by Diacerein (Dia) can suppress angiogenesis/invasion by up-regulating MAO-A expression. This study presents role of IL-6/IL-6R-MAO-A signalling axis in invasion/angiogenesis in hypoxic tumour environment and highlights this signalling as critical target for cancer therapy.

## Materials and method

### Chemicals, reagents, and cell lines

For cell culture, DMEM medium, Sodium bicarbonate, antibiotics, and others were obtained from standard manufactures (Invitrogen, Sigma and Himedia). Various antibodies were purchased from Cell Signaling Technology, Beverly, MA, USA and Santa Cruz Biotechnology, USA. IL-6R siRNA was purchased from Santa Cruz Biotechnology, USA. 5-Azacytidin (5-Aza) and Diacerein (Dia) were purchased from Sigma-Aldrich, St. Louis, MO, USA. Other chemicals were purchased from Sigma-Aldrich, St. Louis, MO, USA and Himedia India. MDA-MB-468 and MDA-MB-231 cells were purchased from National Center for Cell Science, Pune, India. HUVEC cells were purchased from Himedia India.

### Western blot analysis

Breast cancer cells were exposed to different conditions like normoxia, hypoxia (By CoCl2 treatment), and treated with pharmacological agents including 5-Aza and Dia. These treated cells were further subjected to western blot analysis according to earlier reported method.^11^

In another set of experiment, cells were subjected to keep in hypoxic environment and treated with different selective inhibitors like Akt inhibitor (LY294002, 10 µM, and 20 µM), MAPK inhibitor (AG126, 20 µM, and 30 µM) and STAT3 inhibitor (PF04965842, 30 nM, and 50 nM) for 48 h. Then immunoblot analysis was performed to these treated cells.

### Immunofluorescence study of breast cancer cells

MDA-MB-231 and MDA-MB-468 cells were grown on cover slips. After 60–70% confluency, complete medium was removed and incomplete medium was added. Freshly prepared CoCl2 (100 µM) solution was added to the medium to induce hypoxia. After hypoxic induction, cover slips were washed, fixed with 3.4% paraformaldehyde and blocked with 3% BSA. Primary antibodies and FITC tagged secondary antibodies were used to specify the expression of MAO-A and IL-6R. Propidium iodide (PI) or DAPI was used to stain the nucleus.

### Preparation of conditioning medium (CM)

MDA-MB-231 and MDA-MB-468 breast cancer cells were seeded in 60 mm Petri plate. Cells were then transfected with IL-6R siRNA for 24 h and kept in hypoxic condition. In other groups, hypoxic cells were treated with Dia and 5-Aza for 24 h. After 24 h of treatment, medium was replaced with fresh incomplete medium and incubated for another 48 h. These CMs were collected and kept in −80 °C for further experiments.

### Transfection study in cancer cells

Transfection study was performed under different conditions to knockdown specific proteins according to earlier reported method.^11^ In brief, cancer cells were serum starved and transfected with IL-6R siRNA with the help of FuGENE® HD Transfection Reagent according to manufacturer protocols. After transfection, cells were subjected to western blot analysis.

### Gelatin zymography

Gelatin zymography of CMs was performed to evaluate MMP-2/9 expression according to earlier reported method.^12^ In brief, MDA-MB-231 and MDA-MB-468 cells were grown in 60 mm Petri plates and treated with CoCl2 (100 µM for 24 h) solution to create hypoxic condition. Cells were then exposed by IL-6R siRNA, Dia (6 µM or 4 µM for MDA-MB-231 and MDA-MB-468, respectively), and 5-Aza (5 µM). Conditioning medium was collected from different groups and subjected to Gelatin zymography with specific substrate. Images were captured by gel doc.

### Cytoskeleton arrangement

Cytoskeletal rearrangement is the characteristic feature of invasive cells in hypoxic environment during the EMT transition.^13^ In this study, breast cancer MDA-MB-231 and MDA-MB-468 cells were grown on lysine coated cover slips. Then, cells were treated with CoCl2 (100 µM), 5-Aza (5 µM), and Dia (6 µM for MDA-MB-231 and 4 µM for MDA-MB-468). After 24 h treatment, cells were fixed with 3.4% paraformaldehyde solution and stained with rhodamine-phalloidin for visualisation of actin structure.^14^

### Boyden chamber migration assay

Boyden Chamber assay was performed to access invasion capability of breast cancer cells according to earlier reported method with slight modifications.^15^ Breast cancer cells were treated with CoCl2 (100 µM)) and transfected with IL-6R siRNA. Other groups were treated with Dia (6 µM) or 5-Aza (5 µM). These treated cells were placed on the upper chamber of Boyden set up. In lower chamber, VEGF containing medium was added. After 24 h of incubation, upper chamber was taken and non-migrated cells were swapped with cotton plug. Upper chambers were then fixed with methanol and stained with haematoxylin and eosin followed by proper washing. Migrated cells were subjected to bright field microscopy at ×20 magnifications (Carl Zeiss).

### Wound healing assay

In vitro scratch assay or wound healing assay was performed to study cancer cell migration.^16^ In brief, breast cancer cells were seeded in six well plates. After 60–70% confluence, a scratch was drawn with pipette tips. Unattached cells were washed with incomplete medium. Cells were transfected with IL-6R siRNA and treated with Dia and 5-Aza in hypoxic environment. After 48 h, images of scratch area were captured.

### Immunofluorescence study for E-Cad detection

MDA-MB-231 and MDA-MB-468 cells were grown on cover slips in different groups. Then, cells were exposed to CoCl2 for hypoxic induction. Hypoxic cells were treated with IL-6R siRNA, Dia (6 µM or 4 µM for MDA-MB-231 and MDA-MB-468, respectively), and 5-Aza (5 µM) for 48 h. After treatment, cells were fixed with 3.7% paraformaldehyde, blocked with 3% BSA and incubated for overnight with primary antibody (E-Cad). FITC tagged secondary antibodies was added and DAPI was used as counter stain. Florescent images were captured under microscope.

### Colony formation assay

Cell proliferative capacity of cancer cells were measured by colony formation assay according to earlier reported method.^17^ In brief, breast cancer cells were transfected with IL-6R siRNA in hypoxic condition. Other groups were treated with Dia (6 µM or 4 µM for MDA-MB-231 and MDA-MB-468, respectively) or 5-Aza (5 µM) for 48 h. These treated breast cancer cells were collected and seeded in another six well plate (1000 cells per well). These six well plates were incubated for another seven days. After incubation period, plates were taken and stained with crystal violet (0.005%) solution. Images were captured by gel doc system. Arbitrary number of colonies were counted in each group (Cluster of more than 50 cells counted as single colony).

### Chick chorioallantoic membrane (CAM) assay

CAM assay was performed to access pro-angiogenic effect of different CMs collected from different treated groups. 7–9 days fertilised eggs were collected. Window was carefully opened by breaking eggshell. Chorioallontoic membrane was then exposed by sterile filter discs soaked with different CMs. After 24 h, filter discs were removed and images were taken by stereomicroscopes.

### Human umbilical vein endothelial cells (HUVEC) assay

Tube formation assay was performed in HUVEC cells according to earlier reported method.^18^ In brief, HUVEC cells were collected and seeded on matrigel coated 24 well plates. Cells were then treated with different CMs for 24 h. Then medium was removed and stained with calcein AM to stain live HUVEC cells.

### Matrigel plug assay

In vivo blood vessels growth was tested in solidified matrigel implanted in Swiss albino mice according to earlier reported method.^19^ About 300 µL matrigel with recombinant IL-6, 5-Aza (5 µM) and Dia (4 µM) was injected in the flank of mice and allowed to solidify. After 7 days, all mice were sacrificed and solidified matrigels were excised. Images of matrigel plug were captured. Further, matrigel plugs were processed for immunofluorescence (CD-31) assay, as well as haematoxylin and eosin staining was performed.

### Human breast cancer tissue collection

Breast cancer tissue and adjacent normal tissues were collected from breast cancer patients at Calcutta National Medical College, Kolkata, India. Informed consents were obtained from all patients. The study was approved by Institutional human ethics committee of Calcutta National Medical College, Kolkata, India.

### Haematoxylin and Eosin (H&E) staining and immunohistochemistry

Immunohistochemical analysis and H&E staining of tumour samples were performed according to earlier reported method.^20^

### Immunofluorescence of breast cancer tissue

Human breast cancer tissue sections were prepared from paraffin embedded tissue block. Tissue sections were kept at 60 °C to remove paraffin and sections were rehydrated. Immunofluorescence study of tissue sections were performed to detect cellular localisation of IL-6R (Green fluorescence) and MAO-A (Red fluorescence) proteins. DAPI was used as counter stain.^21^

### Software and statistical analysis

Gel-Quant and GraphPad Prism software were used to process experimental results. Each experiment was conducted at least three times. One way ANOVA was performed to predict the significance level. The *p*-value <0.05 was consider to be significant. All the results presented in this article were as mean ± standard deviations.

## Results

### Hypoxia modulated the expression of number of proteins in breast cancer

**Fig. 1:**
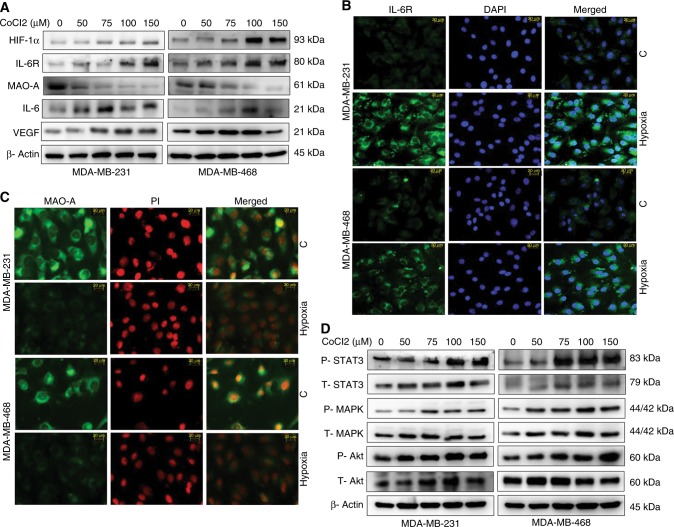
Hypoxia modulates protein expression (**a**) Western blot analysis of MDA-MB-231 and MDA-MB-468 cells in hypoxic condition. Cells were treated with dose dependent CoCl2. Hypoxia increased the expression of HIF-1α, IL-6R, IL-6, and VEGF in both cell lines. Expression of MAO-A was decreased in hypoxic environment. **b** Immunofluorescence study of normoxic and hypoxic breast cancer cells. **c** Expression of MAO-A in normoxic and hypoxic breast cancer cells. **d** Effect of hypoxia on IL-6/IL-6R downstream proteins

MDA-MB-231 and MDA-MB-468 breast cancer cells were exposed with CoCl2 to induce hypoxic environment in dose dependent manner (50, 75, 100, and 150 µM CoCl2) for 48 h. After hypoxic induction cells were subjected to western blot analysis. The expression profiles of a panel of proteins were critically analysed. Hypoxic induction with CoCl2 up-regulated IL-6, IL-6R, HIF-1α, and VEGF expression compare to control in both cells. Interestingly, MAO-A expression was significantly down-regulated in hypoxic cancer cells in dose dependent manner (Fig. 1a and Fig. S1). Further, immunofluorescence study showed high expression of IL-6R and low expression of MAO-A in hypoxic cells compared to the normoxic cells (Fig. 1b, c).

### Activation of IL-6/IL-6R downstream proteins by hypoxia

IL-6 generally acts through activation of three different downstream pathways including STAT3, Akt and MAPK signalling.^22^ In this study, cells were exposed to dose dependent hypoxic conditions and subjected to western blot analysis to check expression of above proteins and phospho status of these proteins (Fig. 1d and Fig. S2A). P-Akt, P-STAT3, and P-MAPK were up-regulated. Expression of total Akt, MAPK, and STAT3 was also changed in different groups (Fig. 1d and Fig. S2B).

### Inhibition of IL-6R with siRNA or Dia up-regulated MAO-A activity

**Fig. 2:**
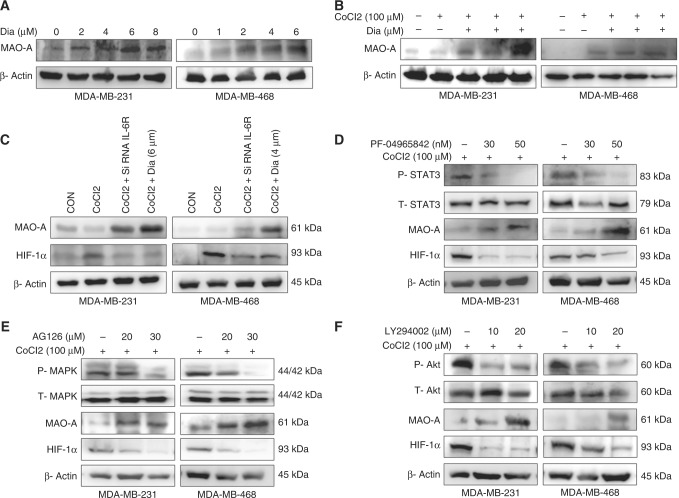
Inhibitory effect in different targets. **a** Effect of IL-6R/IL-6R inhibition by Dia on MAO-A activity by western blot analysis. **b** Effect of IL-6/IL-6R inhibition by Dia treatment in hypoxic breast cancer cells on MAO-A activities. MAO-A expression was highly up-regulated due to inhibition of IL-6. **c** Cells were treated with CoCl2 and siRNA IL-6R transfection or Dia treatment was performed in hypoxic breast cancer cells. MAO-A and HIF-1α expression was shown. **d** Western blot using STAT3 inhibitor (PF-04965842) in dose dependent manner. **e** Western blot using AG126, a specific inhibitor of MAPK. **f** Western blot using LY294002, a specific PI3K/Akt inhibitor

Next, we utilised siRNA of IL-6R and a small molecule Dia that inhibited an IL-6/IL-6R signalling and its downstream pathways.^22^ Dia treatment inhibited phospho level of Akt, MAPK, and STAT3 in hypoxic cells (Fig. S3). In hypoxic condition, as well as normoxic breast cancer cells significantly overexpressed MAO-A after dose dependent treatment of Dia for 48 h (Fig. 2a, b, Figs. S4 and S5). In addition, breast cancer cells were treated with CoCl2 (100 µM) and transfected with IL-6R siRNA (S6A) and treated with Dia. In hypoxic cells, IL-6 siRNA or Dia treatment caused sustained up-regulation of MAO-A (Fig. 2c, Fig. S6B). In contrast, HIF-1α expression was sharply reduced in transfected IL-6 siRNA or Dia exposed breast cancer cells in hypoxic environment.

### Specific inhibition of Akt, STAT3 and MAPK up-regulated MAO-A activity

We used different inhibitor of IL-6/IL-6R downstream pathways to examine which pathway is involved to regulate MAO-A expression by IL-6. Hypoxic breast cancer cells were treated with specific inhibitors of Akt (LY294002), MAPK (AG126), and STAT3 (PF04965842) followed by MAO-A and HIF-1α activities were measured. All the inhibitors potentially increased the expression of MAO-A and inhibited the HIF-1α expression. These findings indicated that IL-6/IL-6R regulates MAO-A via Akt, STAT3 and MAPK pathways (Fig. 2d, e, f, Fig. S7).

### 5-Aza up-regulated MAO-A in breast cancer cells

**Fig. 3:**
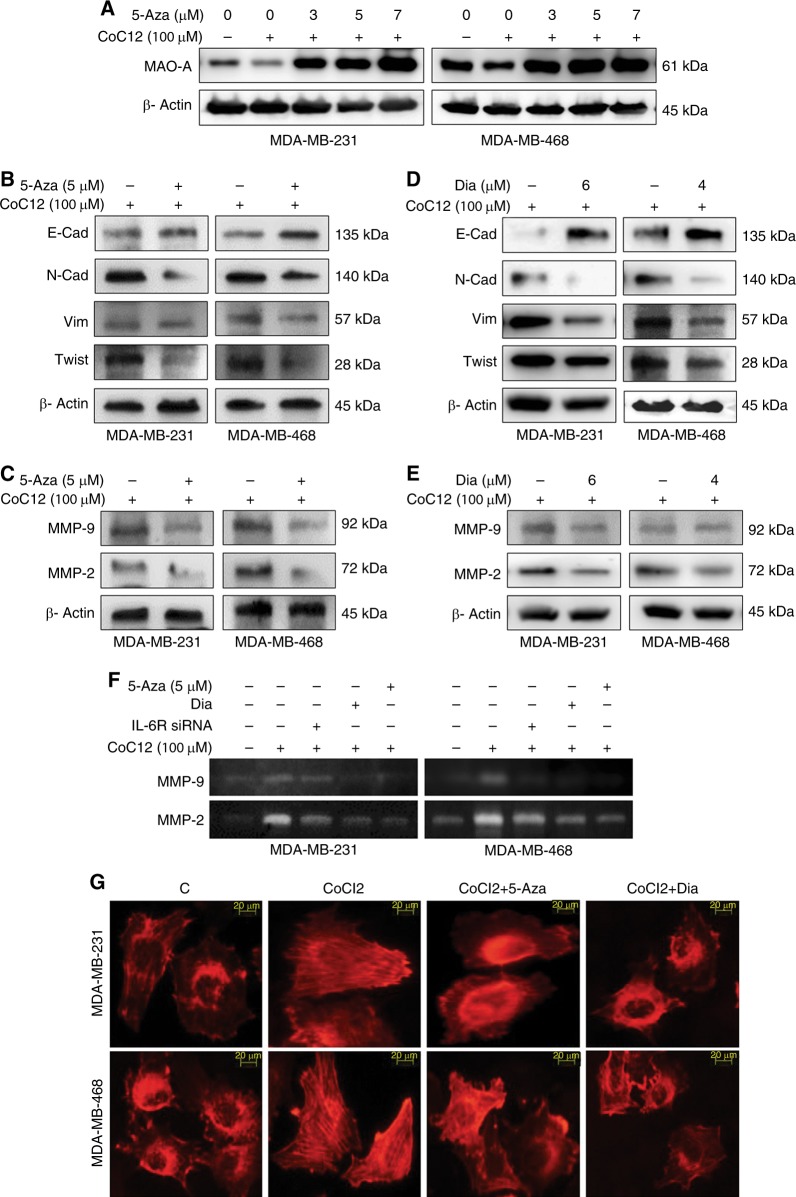
Assessment of EMT and invasion markers. **a** 5-Aza was treated to check the MAO-A expression in hypoxic breast cancer cells. **b** Overexpression of MAO-A was induced by 5-Aza in hypoxic cancer cells. Different EMT markers E-Cad, N-Cad, Vim, and twist were analysed by western blot analysis. **c** Expression of MMP-2/9 was assessed in hypoxic cells treated with 5-Aza. **d**, **e** Effects of IL-6/IL-6R inhibition with Dia on different EMT markers, as well as MMP-2/9 expression were evaluated in hypoxic breast cancer cells. **f** Gelatinolytic effect of MMP-2/9 in different treatment regimens. **g** Actin organisation of breast cancer cells from different treatment plans

Earlier findings showed that 5-Aza treatments can up-regulate MAO-A expression by inhibiting DNA hypermethylation that regulate MAO-A promoter activity.^23^ Here, we treated hypoxic breast cancer cells with dose dependent 5-aza (3, 5, and 7 µM) for 48 h in dose dependent manner. The dose of 5-Aza was selected 5 µM for further experiment (Fig. 3a and Fig. S8).

### Modulation of MAO-A/IL-6R altered the expression of invasive markers

For mechanistic investigation, MAO-A expression was increased by 5-aza (5 µM) treatment in hypoxic breast cancer cells. In 5-Aza treated cells, the expression of cancer associated invasive markers was tested (E-Cadherin, N-Cadherin, Vimentin, Twist, MMP-2, and MMP-9). Epithelial marker, E-Cad was increased significantly due to up-regulation of MAO-A by 5-Aza treatment. In contrast, mesenchymal markers N-Cad, Vim, and Twist were decreased significantly due to 5-Aza exposure (Fig. 3b and Fig. S9). Expression of MMP-2/9 level was also down-regulated (Fig. 3c and Fig. S9). Similar effect was also observed due to IL-6/IL-6R inhibition by Dia treatment in hypoxic breast cancer cells (Fig. 3d, e and Fig. S10).

### IL-6 inhibition/MAO A activation suppressed MMPs activities

For the measurement of MMP-2/9 activities, we performed gelatin zymography assay of CMs. Breast cancer cells were subjected to chemical hypoxia and further transfected with IL-6R siRNA or treated with 5-Aza and Dia. Hypoxia in cancer cells caused up-regulation of MMP-2/9 in both MDA-MB-231 and MDA-MB-468 cancer cells. In contrast the expression of MMP-2/9 was significantly reduced in IL-6 siRNA, Dia, and 5-aza treated groups in both cells. So, IL-6 inhibition or up-regulation of MAO-A can lead to suppression of MMP-2/9 (Fig. 3f).

### Dia/5-Aza treatment modulated the actin arrangement

Breast cancer cells were kept in hypoxic condition and treated with 5-Aza (5 µM) or Dia (6 and 4 µM for MDA-MB-231 and MDA-MB-468, respectively). Actin organisation was evaluated by rhodamine phalloidin staining. Hypoxia caused parallel orientation of actin filaments. Parallel orientation of actin arrangement facilitates cancer invasion. Interestingly, 5-Aza or Dia treatment inhibited parallel orientation of actin filaments (Fig. 3g) that may lead to less invasive behavior.

### IL-6R/MAO-A regulated chemotaxis behavior of cancer cells

**Fig. 4:**
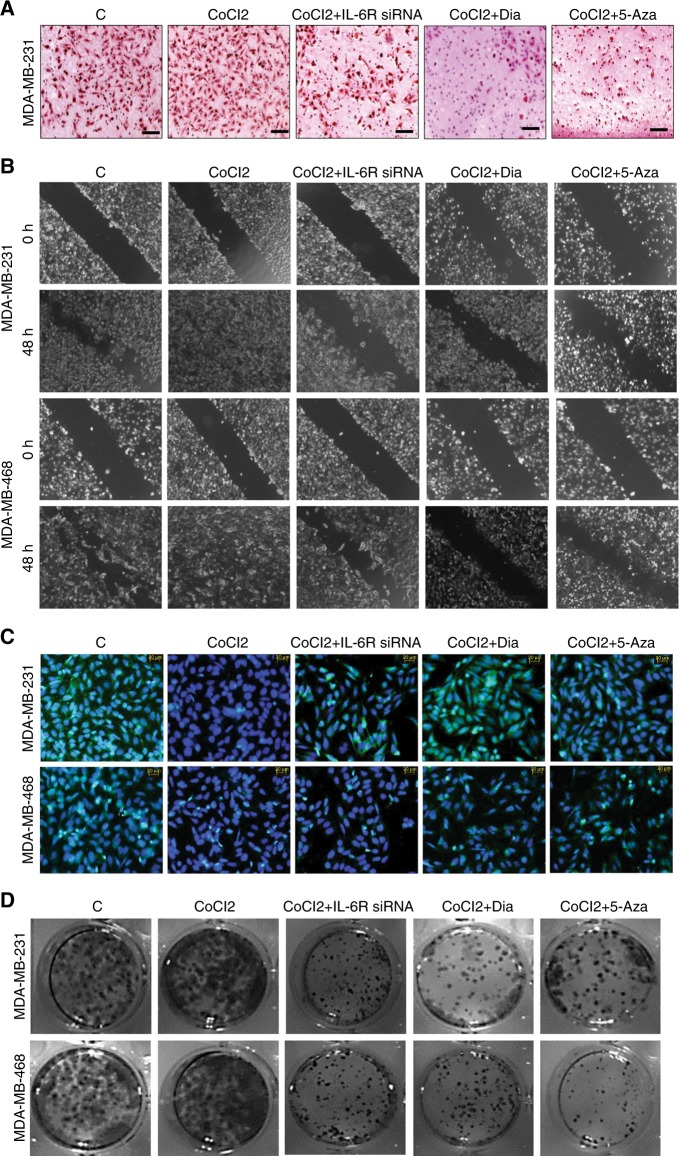
In vitro invasion and migration assay. **a** Boyden chamber assay. **b** Wound healing assay. **c** Immunofluorescence study for detection of E-Cad (Green). Cells were counterstained with DAPI. **d** Colony formation assay

Boyden chamber or chemotaxis assay was performed to capture invasive ability of cancer cells in vitro. We evaluated the invasive nature of cancer cells after inhibition of IL-6R or upregulation of MAO-A by using IL-6R siRNA, Dia and 5-Aza. Hypoxia increased invasive potential of cancer cells. IL-6R siRNA, Dia, and 5-Aza treatment in hypoxic cells significantly suppressed the number of invasive cancer cells (Fig. 4a). Arbitrary quantification of invasive cells from different groups was presented in bar graph (Fig. S11A).

### Modulation of IL-6R/MAO-A changed the cellular migration

Migration properties of breast cancer cells were tested by conventional wound healing assay. CoCl2 treatment in breast cancer cells promoted migration of MDA-MB-231 and MDA-MB-468 breast cancer cells. Interestingly, IL-6R siRNA transfection suppressed cellular migration. Similarly, treatment with Dia or 5-Aza significantly suppressed the migration of breast cancer cells (Fig. 4b, Fig. S11B).

### Hypoxia, IL-6R siRNA, Dia, and 5-Aza treatment modulated the profiling of E-Cad in breast cancer cells

Immunofluorescence of breast cancer cells was performed to evaluate E-Cad expression in hypoxic breast cancer cells and IL-6R siRNA, Dia and 5-Aza treated hypoxic cells. In hypoxia treated groups loss of E-Cad was observed in both MDA-MB-231 and MDA-MB-468 cells compared to normoxic cells. Interestingly, restoration of E-Cad expression was observed in IL-6R siRNA, Dia and 5-Aza treated hypoxic cells (Fig. 4c).

### Hypoxia, IL-6R siRNA, Dia, and 5-Aza treatment modulated the colony forming ability of breast cancer cells

Proliferative ability of cancer cells was detected by colony formation assay. For this purpose cancer cells were exposed to hypoxia. In another group hypoxic cells were treated with IL-6R siRNA, Dia, and 5-Aza for 48 h.These treated cells were seeded in six well plates and incubated for seven days. After that numbers of colony of each group was counted. The number of colonies was more in hypoxic breast cancer cells. In contrast number of colony was significantly reduced in IL-6R siRNA transfected cells. In the same way, the number of colony was drastically reduced in Dia and 5-Aza treated breast cancer cells (Fig. 4d and Fig. S12).

### Hypoxia driven angiogenesis and its modulation by IL-6R and MAO-A

**Fig. 5:**
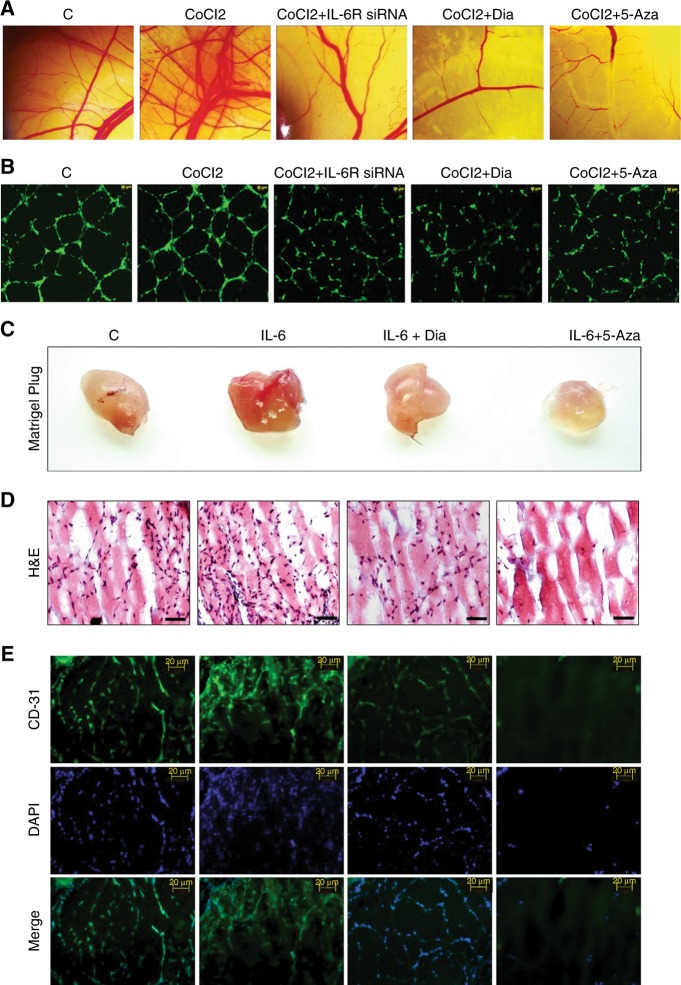
In vitro and in vivo angiogenesis assay. **a** CAM assay. **b** HUVEC or in vitro tube formation assay. **c** Solidified matrigels were excised from the animals after seven days in matrigel plug assay. **d** Matrigel plugs were processed for H&E staining for determination of cellular infiltration inside plugs. **e** Immunofluorescence study of matrigel sections for detection of angiogenesis marker (CD-31)

We performed CAM Assay and in vitro HUVEC assay for assessment of angiogenesis. Hypoxia treated condition medium increased the angiogenic behavior. Other conditioned medium derived from IL-6R siRNA, Dia, and 5-Aza treated hypoxic breast cancer cells potentially suppressed angiogenesis as confirmed by reduced number of micro blood vessels (Fig. 5a and Fig. S13) and tube formation (Fig. 5b and Fig. S14).

### Dia/5-Aza inhibited cellular infiltration and blood vessels growth inside the matrigel

Solidified matrigels from each group were taken out and images of those were captured. In recombinant IL-6 treated group, huge number of infiltrating cells were observed in H&E staining (Fig. 5c, d). However, in Dia or 5-Aza treated group, number of infiltrating cells inside the matrigel were drastically reduced. CD-31(Angiogenesis marker) expression was drastically reduced in Dia or 5-Aza treated groups compared to only recombinant IL-6 treated and control groups (Fig. 5e).

### Gradation of IDC displayed variable expressions of different protein markers

**Fig. 6:**
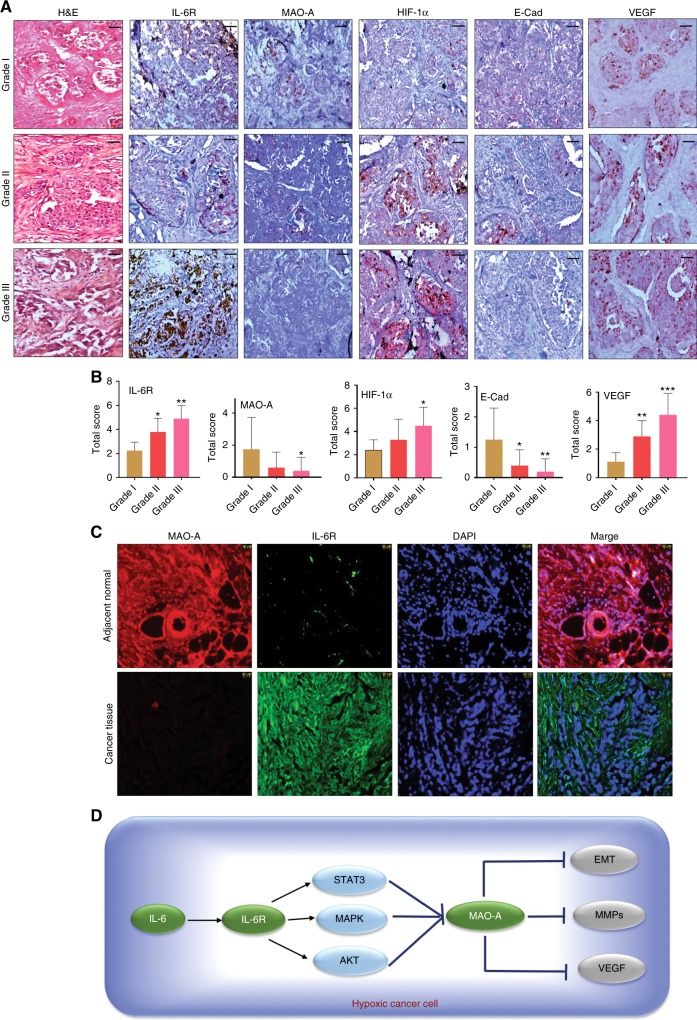
Analysis of clinical breast cancer sample. **a** Grade I, Grade II and Grade III of IDC tissue samples were taken. H&E staining of different grades of cancer was performed. Then, immunohistochemistry of a panel of marker proteins IL-6R, MAO-A, HIF-1α, E-Cad, and VEGF was analysed. Scale bar was 10 µm. **b** Quantification level of staining of IL-6R, MAO-A, HIF-1α, E-Cad, and VEGF. **c** Differential expression of MAO-A and IL-6R. Immunofluorescent detection of MAO-A and IL-6R in high grade IDC and adjacent normal tissue. **d** Graphical representation of hypothesis

We took different grades of tumour tissues from IDC breast cancer patient. The gradation was performed by clinical pathologist in low grade (Grade I), medium grade (Grade II), and high grade (Grade III) based on differentiation, nuclear feature and mitotic activity. This grading was performed according to the guidelines of modified Bloom–Richardson–Elston grading system. Based on H&E staining, gradation was detected in different IDC samples (Fig. 6a). Expression of different marker proteins IL-6R, MAO-A, HIF-1α, E-Cad, and VEGF was tested in corresponding tissue samples. The expression of IL-6R was significantly high in high-grade IDC. On the other hand, MAO-A expression was significantly low in high grade IDC. Mostly, high grade sample had high IL-6R expression and low MAO-A expression. The expression of HIF-1α and VEGF was high in high grade IDC compared to low grade. Loss of E-Cad was observed in high grade carcinoma (Fig. 6a, b). Clinical details were presented in Table S1.

### Localisation study displayed differential expression of IL-6R and MAO-A in clinical samples

We examined the IL-6R and MAO-A expression in cancerous tissue (High grade IDC) and adjacent normal tissue. In adjacent normal area, the expression of MAO-A was very high and IL-6R expression was significantly low in the MAO-A positive area. In contrast, cancerous tissue (High grade IDC) showed very low expression of MAO-A and IL-6R was found to be overexpressed in the same field. DAPI was used as nuclear staining (Fig. 6c).

## Discussion

MAO-A is the mitochondrial protein that commonly degrades various monoamines like norepinephrine, dopamine and serotonin.^24^ Abnormal activity of MAO-A has been studied in details in many neurological disorders.^25,26^ However, few reports were previously documented regarding the role of MAO-A in cancer or its associated events like angiogenesis and invasion. Inhibition of MAO-A with specific pharmacological agent clorgyline can lead to prevention of cellular apoptosis in melanoma cancer cells.^7^ In another report, suppression of MAO-A was observed in cholangiocarcinoma and this phenomenon was correlated to less invasive phenotype.^23^ Earlier findings also suggested that MAO-A is significantly downregulated in multiple cancers including breast carcinoma.^3,4^ Further, Rybaczyk et al. proposed that decrease expression of MAO-A may be indicator of cancer development. However, the deferential role of MAO-A and IL-6R in cancer invasion/angiogenesis was not demonstrated previously. IL-6 is a critical cytokine having a diverse actions on tumour development, invasion, chemoresistance and cancer stem cell formation.^9,27,28^ In this study, we reported that activated IL-6/IL-6R signalling suppressed the MAO-A activity in hypoxic breast cancer. This signalling axis promoted invasion and angiogenesis via EMT induction, activation of MMP-2/9 and VEGF.

In our study, it was found that IL-6/IL-6R protein was amplified in hypoxic condition. In contrast, loss of MAO-A level was observed in hypoxic environment (Fig. 1a, b, c). Downstream of IL-6/IL-6R signalling like P-Akt, P-MAPK and P-STAT3 were assessed in dose dependent hypoxia (Fig. 1d). CoCl2 treatment critically up-regulated P-Akt, P-MAPK and P-STAT3 indicating their involvement in tumour hypoxia. In next, we used different pharmacological inhibitors to validate our hypothesis. Dose dependent Dia (An IL-6/IL-6R signalling inhibitor) treatment was performed in normoxic, as well as hypoxic breast cancer cells. It was observed that inhibition of IL-6/IL-6R caused activation of MAO-A (Fig. 2a, b). It was observed that both IL-6R siRNA, as well as Dia treatment induced MAO-A activity in hypoxic cells (Fig. 2c). We then checked the involvement of IL-6/IL-6R downstream Akt, STAT3, MAPK in regulation of MAO-A activity by employing different pharmacological inhibitors of those proteins.. All inhibitors increased MAO-A activity in hypoxic breast cancer cells (Fig. 2d, e, f). These findings validated that IL-6/IL-6R regulated MAO-A activity through downstream effectors STAT3, MAPK and Akt. In next experiment, we systematically evaluated the consequence of MAO-A overexpression using 5-Aza (MAO-A activator) in hypoxic breast cancer cells. Dose dependent 5-Aza treatment increased MAO-A expression in hypoxic cancer cells (Fig. 3a). Specific treatment of 5-Aza (5 µM) altered the profile of invasion markers. EMT markers (E-Cad, N-Cad, Vim and twist) and MMPs are critically involved in invasive and malignant transformation.^29,30^ Epithelial marker E-Cad was up-regulated and mesenchymal markers N-Cad, Vim and twist were downregulated in both MDA-MB-231 and MDA-MB-468 cells due to 5-Aza treatment (Fig. 3b). Inhibition of IL-6/IL-6R with Dia displayed the similar effect in hypoxic breast cancer cells (Fig. 3d). 5-Aza treatment, as well as Dia treatment inhibited the MMP-2/9 expression in hypoxic breast cancer cells (Fig. 3c, e). Further gelatinolytic activity of MMP-2/9 was decreased as a result of 5-Aza, Dia and IL-6R siRNA treatment compared to only hypoxic cells (Fig. 3f). IL-6 siRNA, Dia, and 5-Aza inhibited cancer cells invasion in Hypoxic environment individually (Fig. 4a). In the same way, this treatment strategy displayed similar effect in cell migration ability of cancer cells (Fig. 4b). Hypoxia potentiated the loss of E-Cad in MDA-MB-231 and MDA-MB-468 cells. Then hypoxic cells treated with IL-6R siRNA, Dia, and 5-Aza restored the E-Cad expression making the cancer cells less invasive (Fig. 4c). The proliferating ability of cancer cells was drastically modulated after IL-6R siRNA, Dia, and 5-Aza treatment. Hypoxic cells displayed high proliferative ability compared to normoxic cells (Fig. 4d). IL-6R siRNA treatment, Dia, and 5-Aza treatment lead to reduced proliferative ability in hypoxic breast cancer cells due to inhibition of IL-6/IL-6R or activation of MAO-A. The malignant transformation occurs in tumour after cellular and molecular collaboration between invasion and angiogenesis.^31,32^ Further, IL-6R siRNA, Dia, and 5-Aza treated conditioned media inhibited angiogenesis as evidenced by CAM and HUVEC assay (Fig. 5a, b). These above findings were corroborated with the results from western blot analysis (Fig. 1a and Fig. S3). Further, matrigel plug assay validated our hypothesis. Recombinant IL-6 treatment increased cellular infiltration and CD-31 expression (Angiogenesis marker) inside the solidified matrigels. Dia and 5-Aza treatment alone with recombinant IL-6 suppressed cellular infiltration and CD-31 expression in matrigel (Fig. 5c, d). Analysing above findings, it can be said that inhibition IL-6R or activation of MAO-A lead to suppression of VEGF in hypoxic breast cancer cells.

Presence of excess IL-6R, as well as significant low MAO-A were observed in high grade IDC of breast cancer (Fig. 6a). This differential expression of IL-6R and MAO-A may influence on invasive and angiogenic signatures including E-Cad and VEGF in IDC. Loss of E-Cad and high VEGF level were observed in high grade carcinoma (Fig. 6 and Table S1). The results from clinical samples of IDC patients confirmed our in vitro findings. In adjacent normal breast tissue, low expression of IL-6R and high MAO-A were in same area of tissue section. On the other hand, high level of IL-6R and almost absence of MAO-A were observed in cancerous area in high grade IDC (Fig. 6c).

Hypoxic environment greatly reshapes the cellular and molecular architectures in tumour. Under the influence of hypoxic stress, angiogenic and invasive signatures are activated leading to development of more aggressive cancer phenotype. IL-6/IL-6R was found to be critically involved in regulation of MAO-A activity. This signalling axis activated cancer invasion and angiogenesis in hypoxic environment via modulating EMT regulators, MMPs and VEGF (Fig. 6d). Finally, inhibition of IL-/IL-6R signalling hampered the tumour invasion and angiogenesis. In conclusion, this study validated the rational for targeting IL-6/IL-6R-MAO-A axis for development of novel therapeutic for breast cancer management.

## Electronic supplementary material

Supplementary file

## Note

This work is published under the standard licence to publish agreement. After 12 months the work will become freely available.
