# Supplementary material for: Differential expression of IL-6/IL-6R and MAO-A regulates invasion/angiogenesis in breast cancer
Source: Br J Cancer. 2018 Apr 26;118(11):1442–52. doi: 10.1038/s41416-018-0078-x (PMC5988749; doi:10.1038/s41416-018-0078-x)
Supplement: Supplementary file 1 — Supplementary file [file 41416_2018_78_MOESM1_ESM.docx]

**Supplementary Information**

**Differential Expression of IL-6/IL-6R and MAO-A Regulates Invasion/Angiogenesis in Breast Cancer**

**Rashmi Bharti^a,1^, Goutam Dey^a,1^, Anjan Kumar Das^b^ and Mahitosh Mandal^a^***

^a^School of Medical Science & Technology, Indian Institute of Technology Kharagpur, Kharagpur-721302, India

^b^Department of Pathology, Calcutta National Medical Collage, Kolkata-70014, West Bengal, India

***Correspondence to:**

**Dr. Mahitosh Mandal**, School of Medical Science and Technology, Indian Institute of Technology, Kharagpur, West Bengal, PIN-721302, India

E-mail: [mahitosh@smst.iitkgp.ernet.in](mailto:mahitosh@smst.iitkgp.ernet.in)

^1^Contribute equally

**S1**
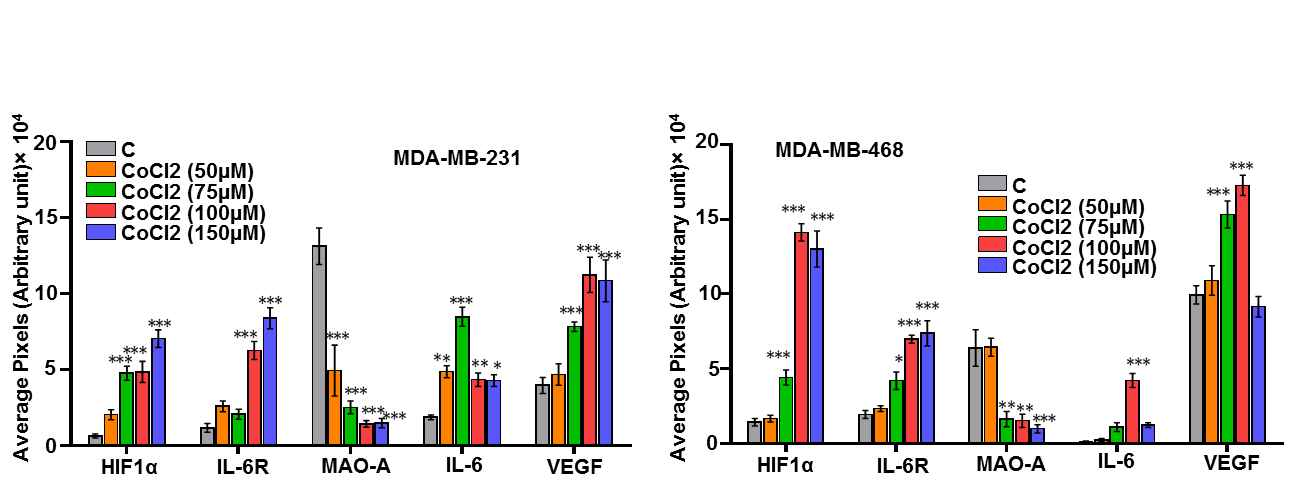
**Figure  S1 Quantative analysis of western blots from Figure 1A**. Comparative analysis of arbitrary expression of different proteins including HIF-1α, IL-6R, MAO-A, IL-6 and VEGF were analysed in MDA-MB-231 and MDA-MB-468 cells. P values less than 0.05 were considered to be significant.

**S2
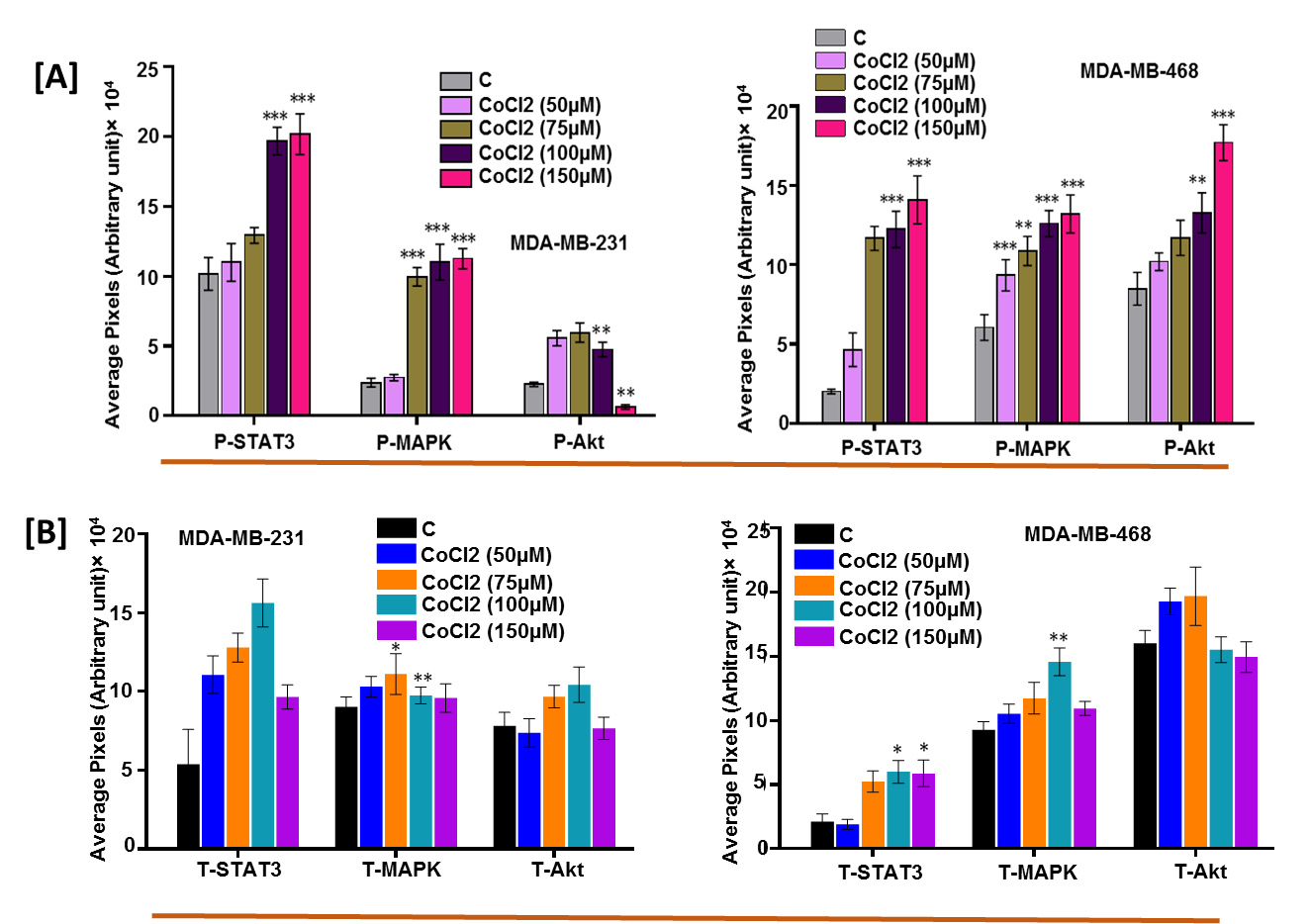
**

**HIF1α**

**VEGF**

**IL-6**

**IL-6R**

**MAO-A**

**Average Pixels (Arbitrary unit)× 10^4^**

**0**

**15**

**20**

**MDA-MB-468**

**5**

**10**


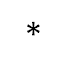

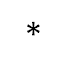

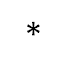

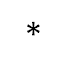

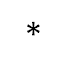

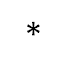

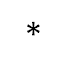

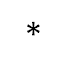

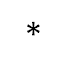

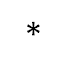

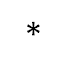

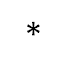

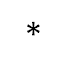

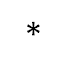

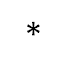

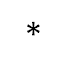

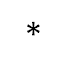

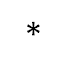

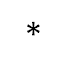

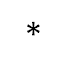

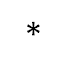

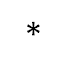

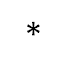

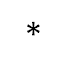

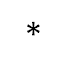


**C**

**CoCl2 (50µM)**

**CoCl2 (75µM)**

**CoCl2 (100µM)**

**CoCl2 (150µM)**

**Figure  S2 Quantative analysis of western blots. [A]** IL-6R downstream proteins P-STAT3, P-MAPK and P-Akt expression were tested in dose dependent CoCl2 exposure [Derived from Figure 1D, Main manuscript**]**. Quantitive expressions of P-STAT3, P-MAPK and P-Akt were presented in bar graph by Gel-Quant image Analysis Software. Expression of protein was analyzed in MDA-MB-231 and MDA-MB-468 cells. **[B]** Quantitive expressions of T-STAT3, T-MAPK and T-Akt were presented in bar graph [Derived from Figure 1D, Main manuscript]. P values less than 0.05 were considered to be significant.

**S3**


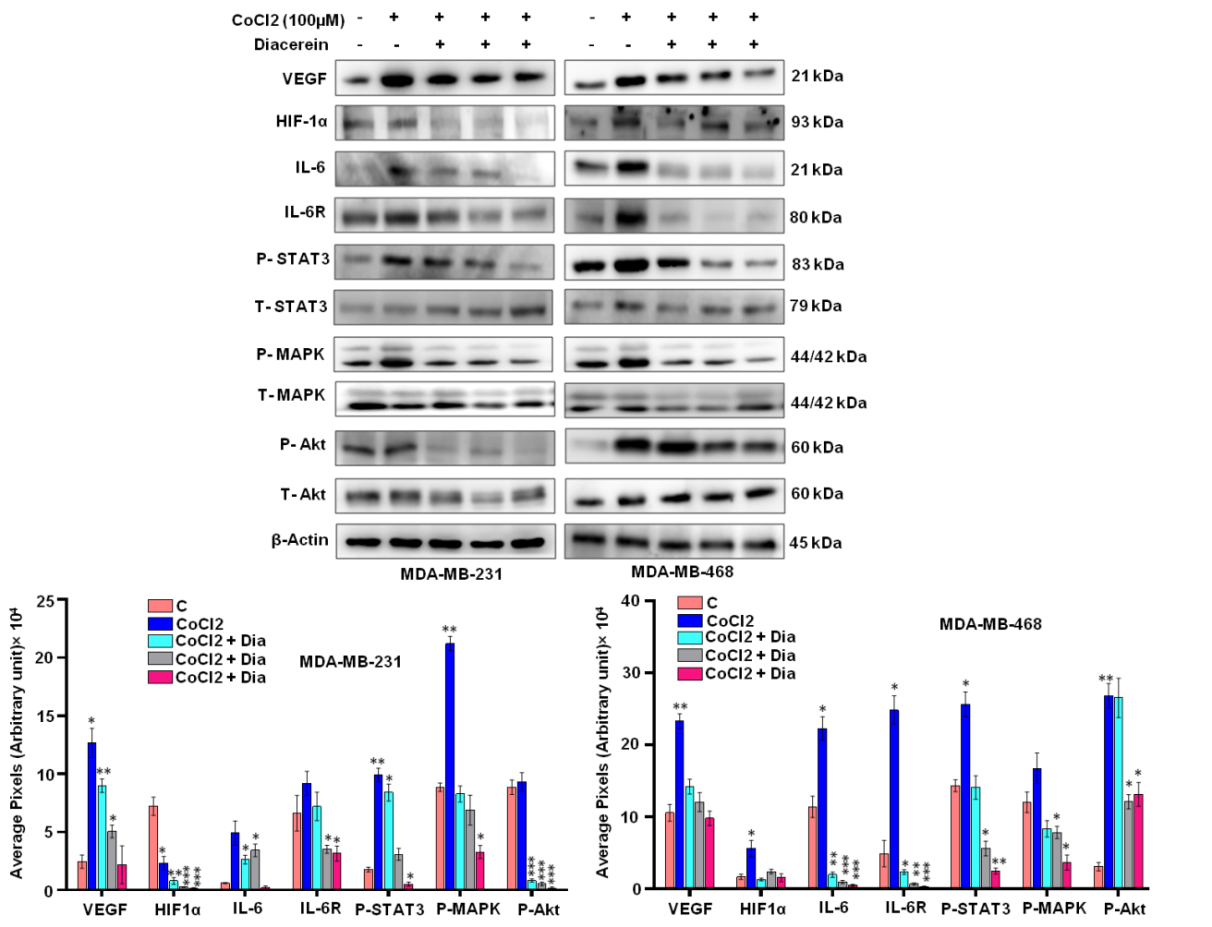


**Figure  S3** Effects of Diacerein were evaluated on the expression of IL-6/IL-6R, its downstream proteins, VEGF and HIF-1α in hypoxic breast cancer cells. Expression of VEGF and HIF-1α was downregulated by diacerein treatment compared to hypoxic cells. Hypoxia promoted overexpression on IL-6/IL-6R and downstreams P-Akt, P-MAPK and P-STAT3. Diacerein treatment in these hypoxic cells caused downregulation of above proteins. Bar graph displayed densitometric analysis of western blots.

**S4**

**
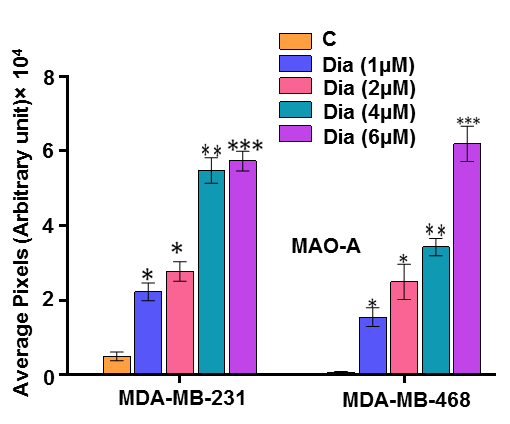
**

**Figure  S4** Dencitometric analysis of western blot derived from Figure 2A (Main manuscript). Quantitive expressions of MAO-A were presented in bar graph by Gel-Quant image analysis Software. Expression of MAO-A was analyzed in MDA-MB-231 and MDA-MB-468 cells treated with dose dependent Dia. P values less than 0.05 were considered to be significant.

**S5**

**
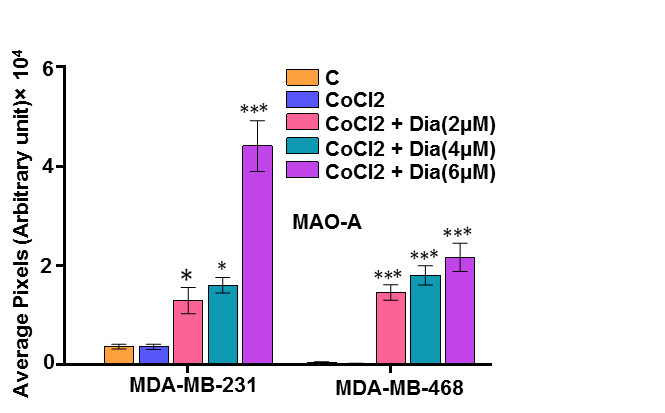
**

**Figure  S5** Dencitometric analysis of western blot derived from Figure 2B (Main manuscript). Quantitive expressions of MAO-A were presented in bar graph by Gel-Quant image analysis Software. Expression of MAO-A was analyzed in MDA-MB-231 and MDA-MB-468 cells treated with dose dependent Dia in hypoxic condition.

**S6**

**
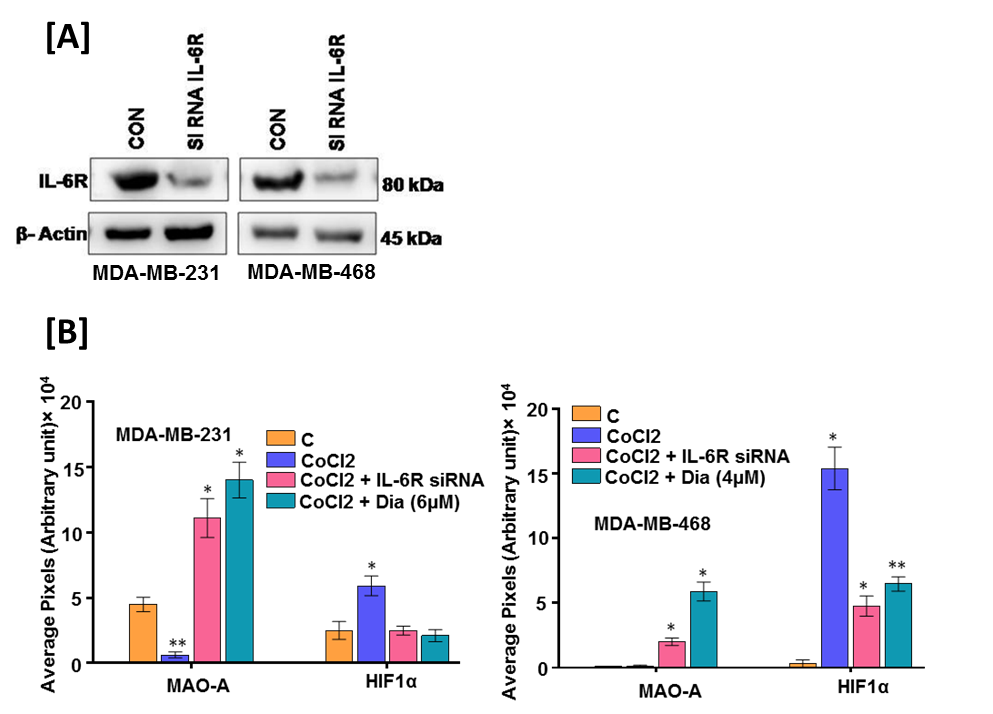
**

**Figure  S6 [A]** Western blot analysis in MDA-MB-231 and MDA-MB-468 cells transfected with IL-6siRNA. **[B]** Dencitometric analysis of western blot derived from Figure 2C (Main manuscript). Quantitive expressions of MAO-A and HIF-1α were presented in bar graph by Gel-Quant image analysis Software. Expression of MAO-A and HIF-1α was analyzed in MDA-MB-231 and MDA-MB-468 cells treated with IL-6R siRNA and Dia in hypoxic condition.

**S7**


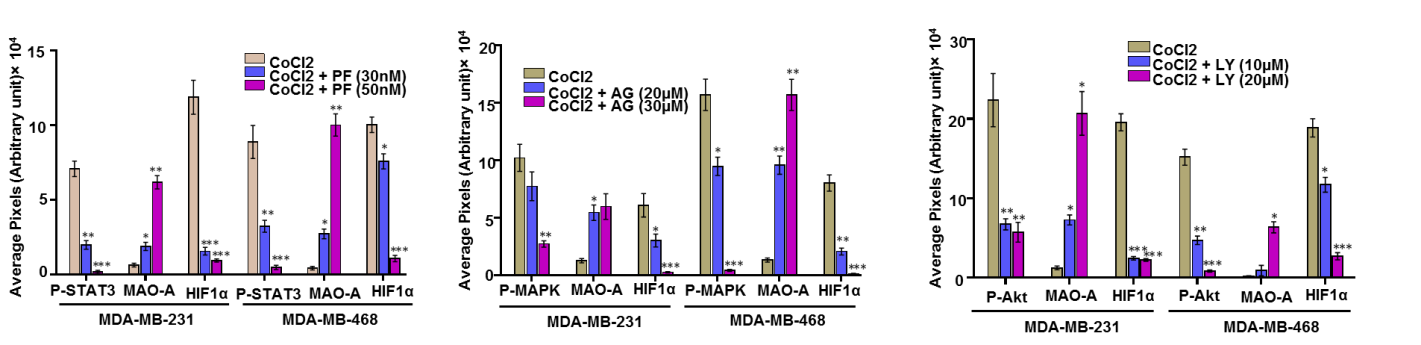


**Figure  S7** Dencitometric analysis of western blot derived from Figure 2D, E, and F (Main manuscript). Quantitive expressions of P-STAT3, P-MAPK, P-Akt, MAO-A and HIF-1α were presented in bar graph by Gel-Quant image analysis Software. Expression of proteins was analyzed in MDA-MB-231 and MDA-MB-468 cells treated with different specific inhibitors (PF04965842-STAT3 inhibitor, AG-126-MAPK inhibitor, and LY294002-Akt inhibitor). P values less than 0.05 were considered to be significant.

**S8**


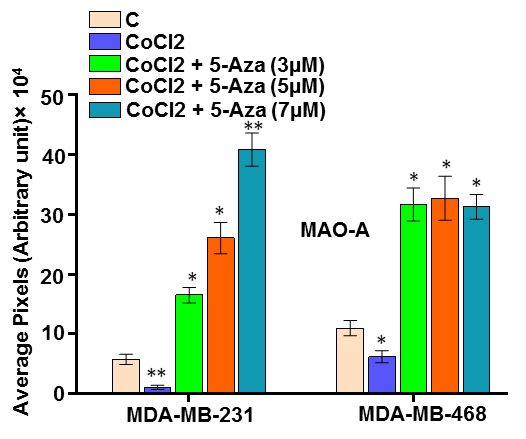

**C**

**CoCl2**

**CoCl2 + Dia**

**CoCl2 + Dia**

**CoCl2 + Dia**

**HIF1α**

**VEGF**

**P-STAT3**

**IL-6**

**IL-6R**

**P-MAPK**

**P-Akt**

**Average Pixels (Arbitrary unit)× 10^4^**

**0**

**10**

**15**

**20**

**5**

**25**

**MDA-MB-231**


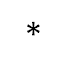

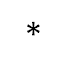

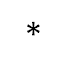

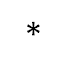

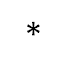

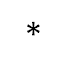

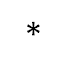

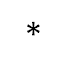

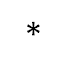

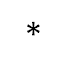

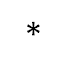

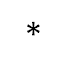

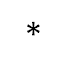

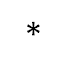

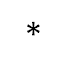

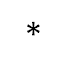

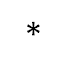

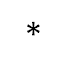

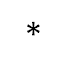

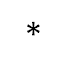

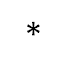

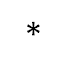

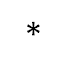

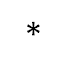

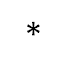

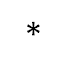

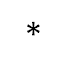

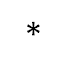

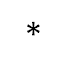

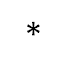

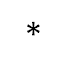

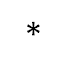

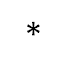


**Figure  S8** Comparative quantitative analysis of densitometric values of each protein expression from figure 3A. P values less than 0.05 were considered to be significant.

**S9**


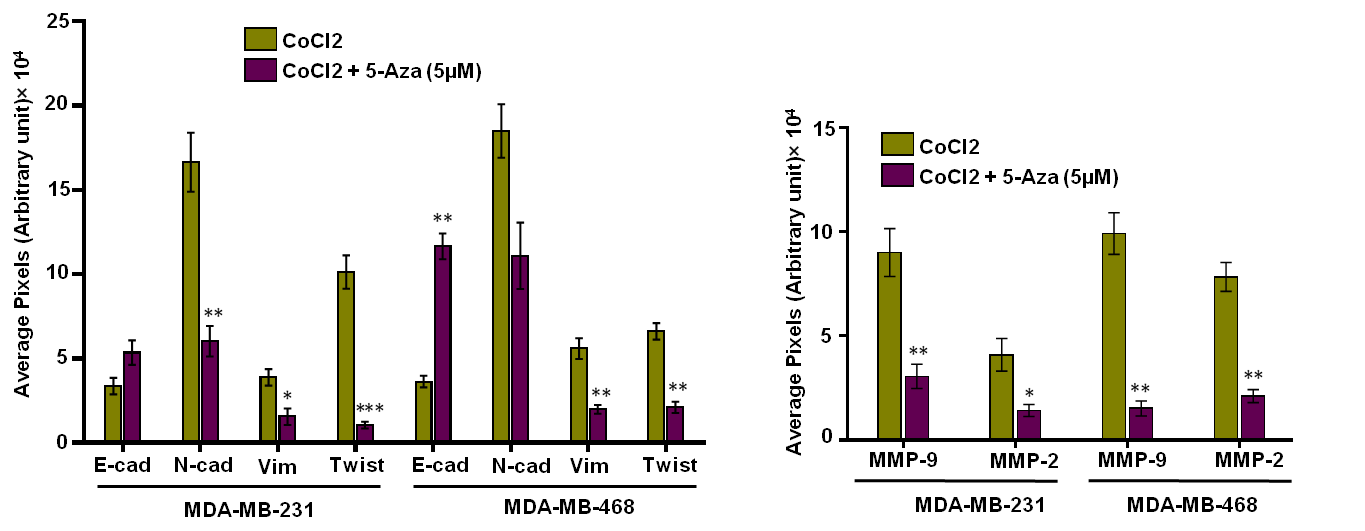


**Figure  S9** Left bar graph indicated densitometric statistics obtained from figure 3B. This bar graph displayed inhibition of N-Cad, Vim and twist and upregulation of E-Cad by 5-Aza treatment in both cell lines. Right bar displayed MMP-2/9 expression that were significantly downregulated after 5-Aza treatment in hypoxic cells (Western blot shown in figure 3C). P values less than 0.05 were considered to be significant.

**S10**


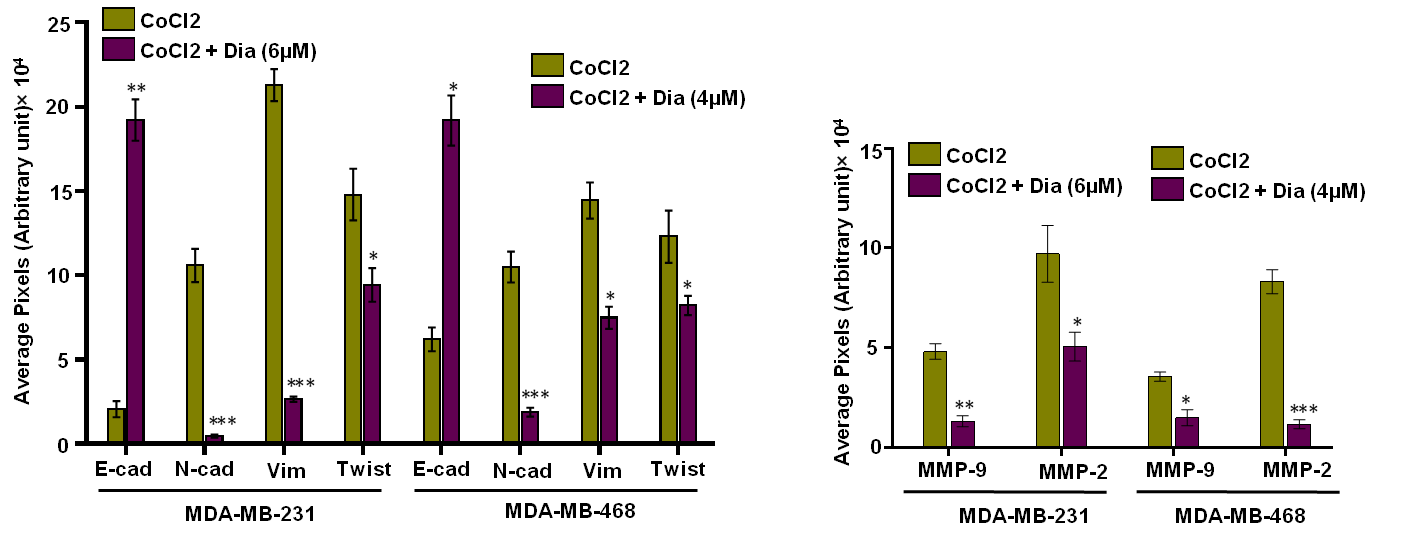


**Figure  S.10** Left bar graph indicated densitometric statistics obtained from figure 3D. This bar graph displayed inhibition of N-Cad, Vim and twist and upregulation of E-Cad by Dia treatment in both cell lines. Right bar displayed MMP-2/9 expression that was significantly downregulated after Dia treatment in hypoxic cells (Western blot shown in figure 3E). P values less than 0.05 were considered to be significant.

**S11**


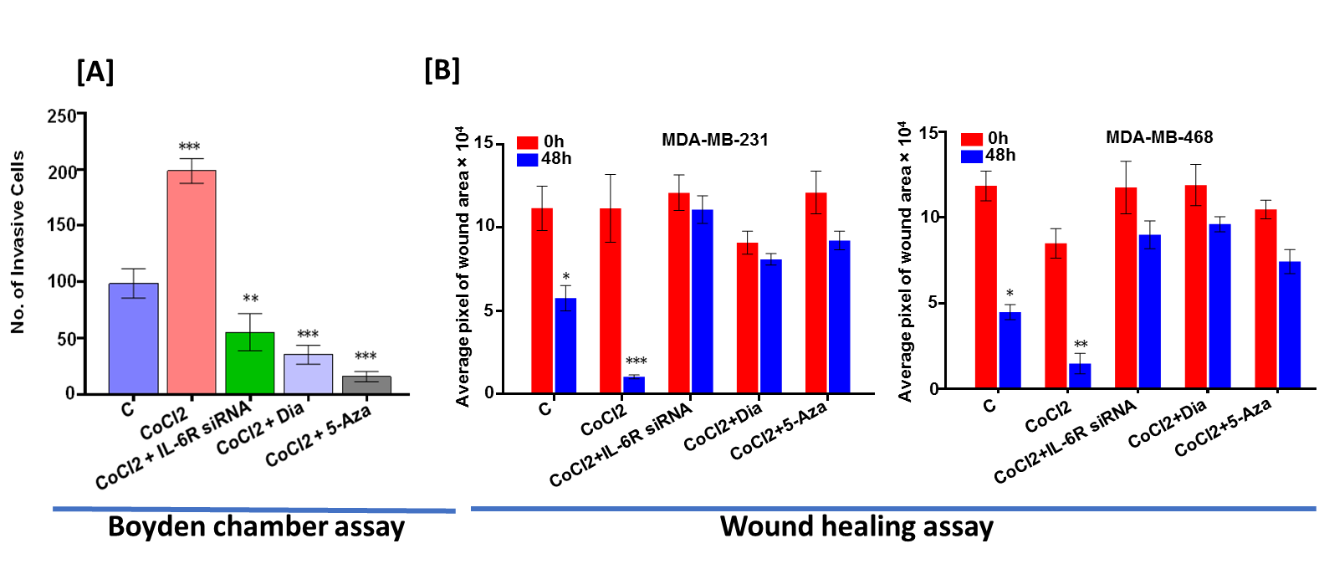


**Figure  S11 Bar graphs. [A]** Boyden Chamber assay (Derived from Figure 4A, main manuscript). Arbitrary number of invasive cells were counted and plotted in bar graph. Most invasive cells were found in hypoxia treated group compare to other groups. IL-6R siRNA, Dia and 5-Aza treated cells in hypoxic group showed significantly less cellular invasion. P values less than 0.05 were considered to be significant. **[B]** Bar graph showing pixel of wound area in different groups. Graphs were derived from Figure 4B (Main manuscript).

**S12**


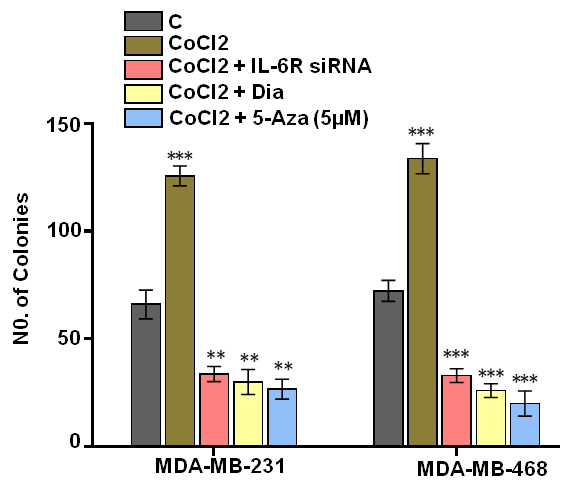


**Figure  S12 Bar graph of colony formation assay was plotted.** Numbers of colonies (Each colony showed at least fifty cells) were counted and plotted in graph. P values less than 0.05 were considered to be significant.

**S13**


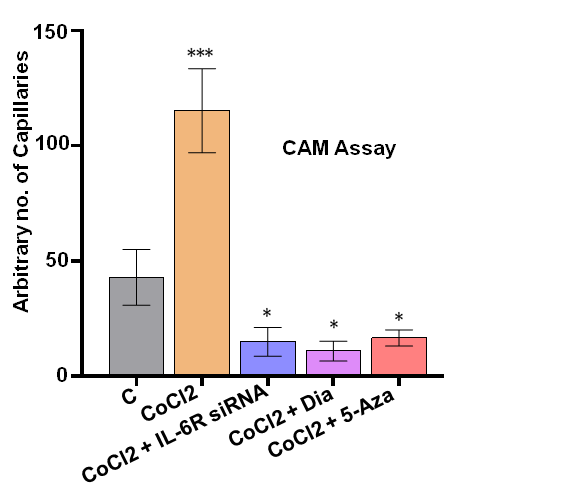


**Figure  S13** Arbitary number of blood vessels (CAM assay, Figure. 5A) were counted and plotted in bar graph. P values less than 0.05 were considered to be significant.

**S14**


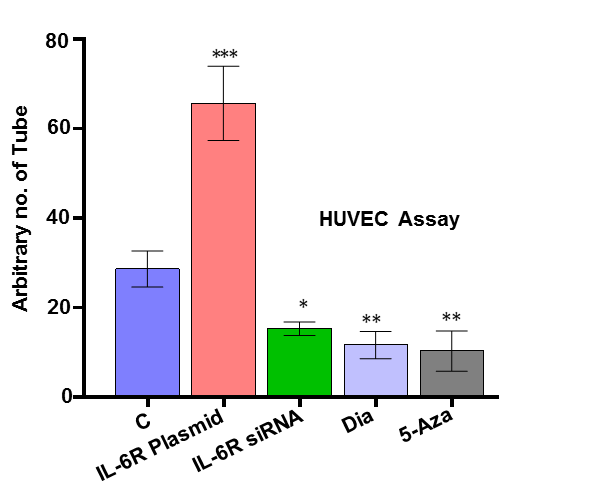


**Figure  S14 Bar graph displayed the arbitrary number of tubes.** Numbers of tube were counted and plotted in the bar graph. Images of HUVEC assay was shown in figure 5B. P values less than 0.05 were considered to be significant.

**Table S1**

Images are quantified by intensity and percentage of positive stain cells as describe below. The intensity of stain was graded 0-3. 0- negative staining, 1- weak staining, 2- moderate staining and 3- high stain.

Percentage of positive stain cell was calculated as follow: 0-10%= 0, 10-30%= 1, 30-50%= 2, 50-70%= 3 and 70-100%= 4.

Total score was calculated by adding scores of intensity and percentage positive cells.

| **No** | **Age** | **Histological Type** | **IL-6R** | **MAO A** | **HIF-1α** | **E-Cad** | **VEGF** |
| --- | --- | --- | --- | --- | --- | --- | --- |
| **Grade-I** |  |  | Total score | | | | |
| 1 | 48 | IDC | 3 | 0 | 1 | 0 | 1 |
| 2 | 52 | IDC | 3 | 2 | 4 | 3 | 2 |
| 3 | 62 | IDC | 3 | 2 | 3 | 1 | 1 |
| 4 | 41 | IDC | 2 | 0 | 2 | 1 | 1 |
| 5 | 65 | IDC | 2 | 1 | 2 | 2 | 1 |
| 6 | 68 | IDC | 2 | 0 | 2 | 0 | 0 |
| 7 | 62 | IDC | 2 | 0 | 2 | 2 | 1 |
| 8 | 59 | IDC | 1 | 2 | 3 | 1 | 2 |
|  |  |  |  |  |  |  |  |
|  |  |  |  |  |  |  |  |
| **Grade-II** |  |  |  |  |  |  |  |
| 1 | 68 | IDC | 3 | 0 | 4 | 1 | 4 |
| 2 | 74 | IDC | 5 | 0 | 5 | 1 | 3 |
| 3 | 71 | IDC | 5 | 1 | 5 | 0 | 2 |
| 4 | 66 | IDC | 2 | 1 | 2 | 0 | 5 |
| 5 | 53 | IDC | 3 | 1 | 1 | 0 | 2 |
| 6 | 55 | IDC | 3 | 0 | 6 | 0 | 3 |
| 7 | 64 | IDC | 3 | 0 | 4 | 0 | 2 |
| 8 | 62 | IDC | 4 | 0 | 2 | 1 | 4 |
| 9 | 49 | IDC | 5 | 0 | 1 | 0 | 2 |
| 10 | 54 | IDC | 5 | 0 | 3 | 1 | 2 |
|  |  |  |  |  |  |  |  |
| **Grade-III** |  |  |  |  |  |  |  |
| 1 | 54 | IDC | 4 | 0 | 6 | 0 | 6 |
| 2 | 72 | IDC | 6 | 0 | 5 | 0 | 6 |
| 3 | 70 | IDC | 5 | 1 | 1 | 0 | 2 |
| 4 | 65 | IDC | 6 | 0 | 4 | 0 | 4 |
| 5 | 61 | IDC | 4 | 0 | 6 | 0 | 2 |
| 6 | 67 | IDC | 6 | 0 | 5 | 0 | 4 |
| 7 | 66 | IDC | 3 | 1 | 5 | 1 | 4 |
| 8 | 57 | IDC | 4 | 0 | 6 | 0 | 5 |
| 9 | 59 | IDC | 5 | 0 | 3 | 0 | 5 |
| 10 | 63 | IDC | 6 | 0 | 4 | 1 | 6 |
